# Neuroimaging distinction between neurological and psychiatric disorders^†^

**DOI:** 10.1192/bjp.bp.114.154393

**Published:** 2015-11

**Authors:** Nicolas A. Crossley, Jessica Scott, Ian Ellison-Wright, Andrea Mechelli

**Affiliations:** **Nicolas A. Crossley**, MRCPsych, PhD, **Jessica Scott**, Department of Psychosis Studies, Institute of Psychiatry, Psychology and Neurosciences, King's College London, London; **Ian Ellison-Wright**, MRCPsych, Avon and Wiltshire Mental Health Partnership NHS Trust, Salisbury; **Andrea Mechelli**, PhD, Department of Psychosis Studies, Institute of Psychiatry, Psychology and Neurosciences, King's College London, London, UK

## Abstract

**Background**

It is unclear to what extent the traditional distinction between neurological and psychiatric disorders reflects biological differences.

**Aims**

To examine neuroimaging evidence for the distinction between neurological and psychiatric disorders.

**Method**

We performed an activation likelihood estimation meta-analysis on voxel-based morphometry studies reporting decreased grey matter in 14 neurological and 10 psychiatric disorders, and compared the regional and network-level alterations for these two classes of disease. In addition, we estimated neuroanatomical heterogeneity within and between the two classes.

**Results**

Basal ganglia, insula, sensorimotor and temporal cortex showed greater impairment in neurological disorders; whereas cingulate, medial frontal, superior frontal and occipital cortex showed greater impairment in psychiatric disorders. The two classes of disorders affected distinct functional networks. Similarity within classes was higher than between classes; furthermore, similarity within class was higher for neurological than psychiatric disorders.

**Conclusions**

From a neuroimaging perspective, neurological and psychiatric disorders represent two distinct classes of disorders.

The ICD-10,^1^ arguably the dominant classification system in use in medicine, makes a distinction between neurological and psychiatric disorders. This distinction is based on nosological criteria, such as aetiological or syndromatic similarities, but also reflects historical^2^ or social factors.^3^ It has important implications for clinical practice, since the remit of services frequently mirror the boundaries between groups of disorders, which might determine the type of treatment individual patients receive.^4,5^ Over the past few years, however, the distinction between psychiatric and neurological disorders has been called into question on the basis of the latest scientific data.^6^ It has been long known that neurological disorders can present with affective or psychotic symptoms traditionally thought to be specific to psychiatric disorders,^7,8^ and that psychiatric disorders present motor symptoms more frequently seen in a neurology clinic.^9^ More recently, brain imaging has provided an *in vivo* window into the human brain, and has revealed that both neurological and psychiatric disorders are associated with neuroanatomical and neurofunctional alterations.^10–12^ This dynamic and efficient perspective on regional changes in brain disorders can complement the histopathological information provided by neuropathological studies.^13^ This approach has challenged the simplistic view of neurological disorders as ‘organic’ and psychiatric disorders as ‘functional’. In addition to neuroscientific evidence, genetic studies have also begun to reveal the genetic underpinnings of neurological and psychiatric disorders. Allelic variants, copy number variants, epistatic effects and gene–environment interactions appear to play a critical role in both classes of disorders,^14–16^ suggesting the presence of comparable aetiological mechanisms. In a recent, thought-provoking article, White and colleagues^6^ suggested that the traditional distinction between disorders of the mind and disorders of the brain is a fundamental misconception, and called for a ‘radical rethinking’ in which psychiatric disorders should be reclassified as disorders of the central nervous system. The merging of these two categories, the authors argued, would be a logical decision given that both neurological and psychiatric disorders are rooted in the brain and are associated with a combination of both sensorimotor and psychological symptoms. However, concerns have been raised about the actual benefits patients would receive from the merging of both fields.^17,18^

We acknowledge that the distinction between the fields of psychiatry and neurology involves multiple factors, ranging from social and historical to biological, and that any new classification should ultimately reflect an improvement in clinical outcomes. However, it is imperative that this debate is informed by scientific evidence including the biology underpinning the two classes of disorders. In this context, we investigated whether neurological and psychiatric disorders have distinct neuroimaging correlates that arguably could reflect distinct neuropathologies. In particular, we examined whether the two classes of disorders affected different sets of regions, whether these regions were localised in different functional networks and whether neuroanatomical variability within each class of disorders is smaller than between classes. Our investigation was based on a meta-analysis of 168 published studies that used structural magnetic resonance imaging (sMRI) to investigate neuroanatomy in a total of 4227 patients and 4504 healthy controls.

## Method

The present meta-analysis was informed by the guidelines provided by the PRISMA (Preferred Reporting Items for Systematic reviews and Meta-Analyses) Statement (http://www.prisma-statement.org/); PRISMA flow diagrams illustrating the number of articles identified for each group of disorders, the number of included and excluded articles, and the reasons for exclusions, can be found in the online Fig. DS1.

### Literature search and selection of studies

In brief, we performed a systematic search for published studies that had employed sMRI and voxel-based morphometry (VBM)^19^ to examine neuroanatomy in patients with a neurological or psychiatric disorder compared with healthy controls. The list of neurological and psychiatric disorders was obtained from chapters V and VI from ICD-10 (2010 version). A total of 91 electronic searches were performed between 2 and 3 May 2012 using the PubMed database. Each electronic search comprised the following general structure: (‘voxel based’ OR morphometr* OR VBM) AND (MRI OR ‘magnetic resonance’) AND terms related to disorder as listed in the ICD-10. When a meta-analysis on a certain neurological or psychiatric disorder was found, we checked the reference list for any studies that had not been detected using our search terms. Some disorders had been examined in only a few VBM studies, whereas others had been examined in a large number of studies. We sought a pragmatic compromise between the need to include as many studies as possible to improve precision of each disorder-specific meta-analysis, and the requirement to include as many disorders as possible to obtain a representative sample of each class. This resulted in the selection of 24 different disorders that had been investigated in at least seven VBM studies (Table 1, and see online Fig. DS1 for a flow diagram of study selection). We classified disorders described in chapter V of the ICD-10 as psychiatric, and those described in chapter VI as neurological. We acknowledge that a number of disorders, in particular the dementias, are included in both chapters, and therefore could be classified either as neurological or psychiatric. For the purpose of the present investigation, we classified neurodegenerative disorders as neurological; we then performed confirmatory analyses to examine how classifying dementias as psychiatric would have an impact on the results.

**Table 1:** List of neurological and psychiatric disorders examined in the present investigation^a^

	Studies included/ published, *n*	Patients/ controls included, *n*
Neurological disorders		
Amyotrophic lateral sclerosis	7/8	114/121
Dementia in Alzheimer's disease	7/36	114/122
Dementia in Parkinson's	7/10	133/172
Developmental dyslexia	7/8	109/108
Dystonia	7/10	151/160
Frontotemporal dementia	7/37	158/170
Hereditary ataxia	7/15	97/121
Huntington's disease	7/9	206/165
Juvenile myoclonic epilepsy	7/7	220/218
Multiple sclerosis	7/11	335/179
Parkinson's disease	7/17	216/197
Progressive supranuclear palsy	7/7	108/182
Temporal lobe epilepsy – left	7/14	232/334
Temporal lobe epilepsy – right	7/10	196/246
Psychiatric disorders		
Attention-deficit hyperactivity disorder	7/13	245/214
Anorexia nervosa	7/10	108/130
Autism	7/12	132/129
Asperger syndrome	7/9	135/177
Bipolar affective disorder	7/18	234/270
Depressive disorder	7/24	146/205
Obsessive–compulsive disorder	7/14	236/211
Panic disorder	7/7	142/133
Post-traumatic stress disorder	7/14	128/126
Schizophrenia	7/51	332/414

a. For each disorder, we report the number of included and published studies and the total number of patients and healthy controls in the included studies. See Online supplement DS1 for details of the included studies.

From each study we extracted the coordinates for grey matter decreases detected in patients relative to controls using a statistical threshold of either *P*<0.05 (whole-brain corrected) or *P*<0.001 (uncorrected). Data were extracted independently by two researchers (N.A.C., J.S.) and any discrepancies resolved by consensus. Coordinates reported in Talairach space were transformed into Montreal Neurological Institute (MNI) coordinates.^20^

### Meta-analysis

In order to obtain a representative picture of the regions affected in neurological and psychiatric disorders, respectively, it was critical that every disorder within its class weighed the same in the final summary. This was ensured in two ways. First, we included the same number of studies per disorder (i.e. seven); if a disorder had been studied in more than seven studies, then the studies included in our investigation were selected randomly. However, the average sample size tended to be larger for those disorders investigated in a greater number of studies (for example schizophrenia) than those investigated in a smaller number of studies (for example panic disorder). This means that a random sample of seven studies for each disorder would still result in different disorders having more or less influence on the results, depending on the sample size of the individual studies. To control for this, we applied a disorder-specific weight to each of the studies included, so that the sum of the weighted sample sizes was equal across disorders. We should highlight that, using this approach, larger studies would still weigh more than smaller studies within each disorder.

Selected studies from each disorder were meta-analysed using the activation likelihood estimation (ALE) method as implemented in GingerALE software (www.brainmap.org/ale),^21^ using a *P*-value of 0.05 (false-discovery rate corrected) and a cluster size threshold of 200 mm^3^. This method models the peak structural differences taking into account the between-subject variance, but also considers empirically informed between-laboratory variance. The comparison between the two classes of disorders was performed using the ALE subtraction analysis;^22^ this involves comparing the difference between the two ALE maps against a null distribution of differences of two similarly sized groups of studies built from random permutation (5000 iterations). Differences between the two classes of disorders were identified using *P*<0.05 (false-discovery rate corrected) and a cluster size threshold of 200 mm^3^.

### Characterisation of network-level brain abnormalities

Any significant abnormalities were characterised by mapping them onto functional networks obtained from a previous investigation using independent component analysis (ICA).^23^ Readers are referred to the original reference for further details on these networks, which have been made available to the neuroimaging community as ‘masks’ by the authors. We also report the anatomical coordinates of the peak weights for each network in online Table DS1). By examining the ratio between number of affected voxels within a specific network and expected number of affected voxels in that network, we were able to establish whether the number of abnormal voxels were randomly distributed across the different networks. To test whether psychiatric or neurological disorders affected differentially ICA-defined brain networks, we compared the difference between their ratios to a null model based on permutation tests. We first randomly permuted the group label (psychiatric or neurological) of the included disorders, resulting in two new ‘random’ groups of psychiatric and neurological disorders. After meta-analysing them individually, we calculated the difference between their ratio of affected voxels within each ICA network. This process was repeated 100 times. Statistical inferences were then obtained by comparing the observed difference in each ICA network to this null-model (one-tailed).

### Estimating heterogeneity within and between classes

In order to characterise the heterogeneity of the two classes of disorders, we computed neuroanatomical variability within each class and between classes. We first meta-analysed each single disorder and computed a measure of similarity between every pair of disorders. This measure was based on the Jaccard index, which is equal to the overlap (intersection) of abnormal voxels normalised by the union of abnormal voxels (i.e. voxels abnormal in both disorders). We then compared the similarity within neurological disorders against that within psychiatric disorders, as well as the similarity within each class against that between classes.

## Results

### Neuroanatomy of neurological and psychiatric disorders

**Fig. 1:**
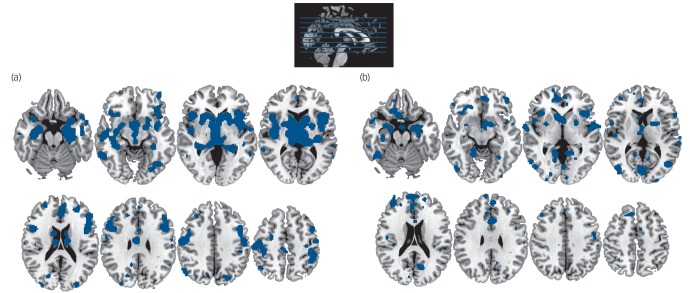
Areas affected in neurological disorders (a) and psychiatric disorders (b) (P<0.05 false-discovery rate corrected).

We first identified those regions consistently affected in neurological and psychiatric disorders separately. As shown in Fig. 1, neurological disorders affected a widespread network comprising the caudate, thalamus, hippocampus, insula, anterior cingulate and sensorimotor cortex bilaterally (see online Table DS2 for details); similarly, psychiatric disorders affected a bilateral network of regions comprising the caudate, hippocampus, insula and anterior cingulate (online Table DS3). Classifying dementing illnesses (Alzheimer's, frontotemporal and dementia in Parkinson's Disease) as psychiatric rather than neurological did not change the overall pattern of results; however, it did result in noticeable changes throughout the temporal cortex. This area of the cortex was primarily implicated in neurological disorders when dementias were classified as neurological, whereas it was primarily implicated in psychiatric disorders when dementias were classified as psychiatric (online Fig. DS2).

**Fig. 2:**
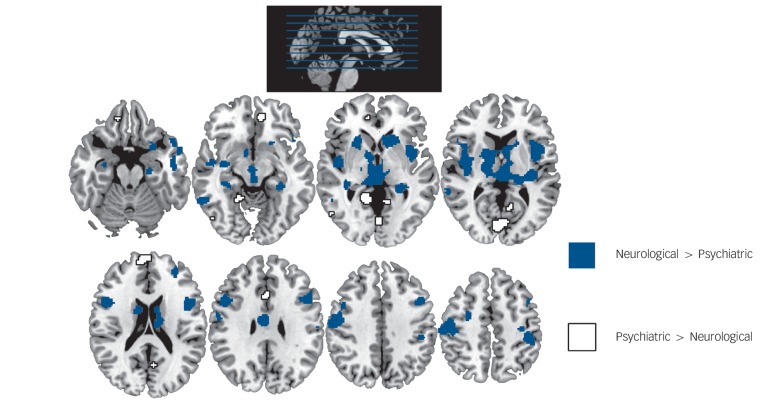
Differential abnormalities between neurological and psychiatric disorders (P<0.05 false-discovery rate corrected).

We then identified regions showing different alterations in neurological and psychiatric disorders by directly comparing the two classes of disorders. As shown in Fig. 2, neurological disorders affected a number of regions more than psychiatric disorders did, including the basal ganglia (thalamus, caudate, putamen and globus pallidus), insula, lateral and medial temporal cortex (including the hippocampus), and sensorimotor areas. Psychiatric relative to neurological disorders showed a more restricted range of abnormalities, located in the medial frontal cortex, anterior and posterior cingulate, superior frontal gyrus and occipital cortex (bilateral lingual gyrus and left cuneus).

### Network-level brain abnormalities

**Fig. 3:**
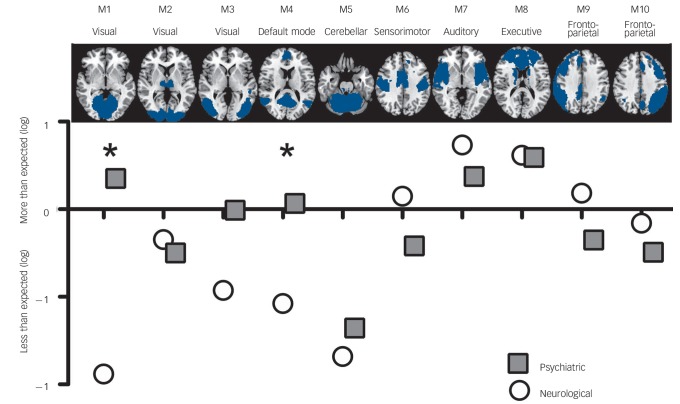
Network fingerprint for neurological (white) and psychiatric (grey) disorders. This figure illustrates the distribution of neuroimaging abnormalities across networks for psychiatric and neurological disorders respectively. In particular, it shows whether psychiatric or neurological disorders affect each of our ten networks of interest more or less than expected (based on the total number of affected voxels). Values correspond to the logarithm of the ratio between observed and expected, with values below zero denoting that abnormalities are less frequent than expected and values above zero denoting that abnormalities are more frequent than expected. The asterisk indicates a statistically significant difference between the two classes at *P*<0.05 (one-tailed permutation tests).

We proceeded by mapping significant abnormalities onto networks obtained from ICA. Specifically, we explored the distribution of abnormalities in each class of disorders among ten well-known networks obtained from ICA from resting state functional MRI data.^23^ This additional analysis provided us with information about which functional networks are disproportionately targeted by each group of disorder (for example how many more/less voxels are abnormal in a network compared with the expected number if the distribution of lesions were homogeneously spread across different networks). This revealed that both classes of disorders affected the auditory temporal network (M7), which includes language areas, and the frontal executive control network (M8), which includes cingulate and paracingulate regions, more than expected. By contrast, both neurological and psychiatric disorders tend to affect the cerebellar (M5) network less than expected. Figure 3 shows that neurological disorders appeared to affect the sensorimotor network (M6) and frontoparietal network (M9) more than psychiatric disorders. By contrast, psychiatric disorders appeared to affect visual networks (M1 and M3) and the default mode network (M4) more than neurological disorders. Permutation tests showed that the two classes of disorders significantly differed in the visual cortex (M1) and default-mode network (M4) (*P*<0.01 and *P* = 0.04, respectively, one-tailed permutation test). This was mostly driven by neurology disorders affecting these networks less than was expected.

### Heterogeneity within and between classes

We also estimated the neuroanatomical heterogeneity within and between classes (online Fig. DS3). The degree of similarity within each class of disorders was higher than the degree of similarity between classes (*P*<0.015, *t*-test). In addition, the degree of similarity was higher for neurological than psychiatric disorders (*P*<10^−4^, *t*-test).

## Discussion

### Main findings

The way in which clusters of symptoms are grouped into different disorders is an important clinical issue, as it determines both diagnosis and treatment of individual patients. The aim of our investigation was to contribute in this discussion by examining whether disorders currently classified as ‘neurological’ and ‘psychiatric’ have distinct neuroimaging correlates. We found that both types of disorders were associated with widespread alterations in cortical and subcortical areas (Fig. 1). In a previous study using a similar approach, we showed that this similarity is driven by the network organisation of the brain.^24^ This observation challenges the traditional distinction between disorders of the mind and disorders of the brain, and provides fresh support for a new conceptual framework in which both neurological and psychiatric disease are considered ‘disorders of the nervous system’.^6^ Although there were many similar brain regions affected across types of disorders, our meta-analytic techniques also showed differences between the two groups of disorders. The basal ganglia, insula, lateral and medial temporal cortex, and sensorimotor areas showed greater impairment in neurological disorders; whereas the medial frontal cortex, anterior and posterior cingulate, superior frontal gyrus and occipital cortex (bilateral lingual gyrus and left cuneus) showed greater impairment in psychiatric disorders. These structural differences between the two classes of disorders affected distinct functional networks, with the effect of neurological disorders evident in the sensorimotor and frontoparietal networks and the effect of psychiatric disorders evident in the visual and default mode networks. Although many of these structural differences are consistent with our existing knowledge of neurological and psychiatric disorders, the greater effect of psychiatric than neurological disorders in visual areas might be surprising to some readers. Closer inspection of the data indicated that this difference was mostly driven by neurological disorders affecting these areas less than was expected based on the total number of significant voxels (Fig. 3). By contrast, abnormalities in occipital areas have often been detected in studies of post-traumatic stress disorder^25^ or schizophrenia.^26^

Disorders within each class, either neurological or psychiatric, were more similar to each other in terms of neuroanatomical alterations than disorders belonging to different classes. In addition, psychiatric disorders were more dissimilar than neurological disorders, speaking of a more heterogeneous class. Taken collectively, these results provide some neuroimaging evidence for the existing distinction between neurological and psychiatric disorders as separate classes of disease. Although such neuroimaging evidence does not necessarily mean that the existing distinction is useful from a clinical perspective, it may inform the current debate on whether the current system should be reconsidered.^6^

The observation of neuroimaging differences between neurological and psychiatric disorders was based on group-level statistical inferences; this raises the question of whether it might be possible to use multivariate statistical learning techniques to identify individual disorders as neurological or psychiatric. We performed an exploratory analysis using a multivariate statistical learning technique known as support vector machine;^27^ this, however, did not yield any significant findings, suggesting that group-level differences do not necessarily allow accurate inferences at the level of the individual disorder. This might be as a result of the high degree of neuroanatomical heterogeneity within each class or, alternatively, a suboptimal methodological approach. In particular, we attempted to classify individual disorders by modelling neuroanatomical abnormalities as spheres centred on the peak coordinates reported by the individual studies, without taking the heterogeneous spatial extent of these abnormalities into account.

### Limitations

The present investigation has several limitations. First, our results might suffer from a selection bias.^28^ In particular, the inclusion of neurological or psychiatric disorders that had been examined in more than seven VBM studies may not have resulted in a representative random sample of each group of disorders. We tried to overcome this limitation by including as many disorders as possible in order to increase the level of representativeness within each class. Similarly, we included disorders with seven or more VBM studies. As previously described, we selected this number based on a compromise between trying to maximise the number of different disorders included, and the precision of the neuroimaging estimate of each disorders. Second, the ALE meta-analysis is based on the frequency with which an effect has been found with a selected statistical threshold, but does not consider variability in the effect size across studies.^29^ Third, we compared the two classes of disorders in terms of grey matter volume only. Ideally, any biologically informed classification of disease should be based on multiple domains including, for example, both brain structure and function, and should use multiple approaches such as neuroimaging, genetics and pharmacology. Finally, we did not consider the effects of age and gender in the different disorders. However, we note that the original VBM studies typically used patient and control groups that were balanced according to age and gender; this means that our results are unlikely to be a result of these confounding variables.

In conclusion, we have shown some divergent neuroimaging findings in neurological and psychiatric disorders; this suggests that neurological and psychiatric disorders represent two distinct classes of disorders from a neuroimaging perspective.

## References

[1] World Health Organization *The ICD-10 Classification of Mental and Behavioural Disorders: Clinical Descriptions and Diagnostic Guidelines*. WHO, 1992.

[2] Martin JB The integration of neurology, psychiatry, and neuroscience in the 21st century. *Am J Psychiatry* 2002; 159: 695–704. 10.1176/appi.ajp.159.5.695 11986119

[3] Baerlocher MO Detsky AS Professional monopolies in medicine. *JAMA* 2009; 301: 858–60. 10.1001/jama.2009.223 19244193

[4] Butler MA Corboy JR Filley CM How the conflict between American psychiatry and neurology delayed the appreciation of cognitive dysfunction in multiple sclerosis. *Neuropsychol Rev* 2009; 19: 399–410. 10.1007/s11065-009-9089-y 19373561

[5] Kanner AM When did neurologists and psychiatrists stop talking to each other? *Epilepsy Behav* 2003; 4: 597–601. 10.1016/j.yebeh.2003.09.013 14698691

[6] White PD Rickards H Zeman AZ Time to end the distinction between mental and neurological illnesses. *BMJ* 2012; 344: e3454. 10.1136/bmj.e3454 22628005

[7] Aarsland D Pahlhagen S Ballard CG Ehrt U Svenningsson P Depression in Parkinson disease–epidemiology, mechanisms and management. *Nat Rev Neurol* 2012; 8: 35–47. 10.1038/nrneurol.2011.189 22198405

[8] Nadkarni S Arnedo V Devinsky O Psychosis in epilepsy patients. *Epilepsia* 2007; 48 (suppl 9): 17–9. 10.1111/j.1528-1167.2007.01394.x 18047594

[9] Fenton WS Prevalence of spontaneous dyskinesia in schizophrenia. *J Clin Psychiatry* 2000; 61 (suppl 4): 10–4. 10739325

[10] Wright IC Rabe-Hesketh S Woodruff PW David AS Murray RM Bullmore ET Meta-analysis of regional brain volumes in schizophrenia. *Am J Psychiatry* 2000; 157: 16–25. 10.1176/ajp.157.1.16 10618008

[11] Kempton MJ Geddes JR Ettinger U Williams SC Grasby PM Meta-analysis, database, and meta-regression of 98 structural imaging studies in bipolar disorder. *Arch Gen Psychiatry* 2008; 65: 1017–32. 10.1001/archpsyc.65.9.1017 18762588

[12] Gong Q Li L Tognin S Wu Q Pettersson-Yeo W Lui S Using structural neuroanatomy to identify trauma survivors with and without post-traumatic stress disorder at the individual level. *Psychol Med* 2014; 44: 195–203. 10.1017/S0033291713000561 PMC3854554 23551879

[13] Fornito A Yucel M Pantelis C Reconciling neuroimaging and neuropathological findings in schizophrenia and bipolar disorder. *Curr Opin Psychiatry* 2009; 22: 312–9. 10.1097/YCO.0b013e32832a1353 19365187

[14] A novel gene containing a trinucleotide repeat that is expanded and unstable on Huntington's disease chromosomes. The Huntington's Disease Collaborative Research Group. *Cell* 1993; 72: 971–83. 10.1016/0092-8674(93)90585-e 8458085

[15] Malhotra D Sebat J CNVs: harbingers of a rare variant revolution in psychiatric genetics. *Cell* 2012; 148: 1223–41. 10.1016/j.cell.2012.02.039 PMC3351385 22424231

[16] Tsuji S The neurogenomics view of neurological diseases. *JAMA Neurol* 2013; 70: 689–94. 10.1001/jamaneurol.2013.734 23571861

[17] Holmes J Minding the brain. *BMJ* 2012; 345: e4581. 10.1136/bmj.e4581 22777552

[18] Bailey S Burn W Craddock N Mynors-Wallis L Tyrer P Suggested merger of mental and neurological illnesses is premature. *BMJ* 2012; 345: e4577. 10.1136/bmj.e4577 22777551

[19] Ashburner J Friston KJ Voxel-based morphometry–the methods. *NeuroImage* 2000; 11: 805–21. 10.1006/nimg.2000.0582 10860804

[20] Lancaster JL Tordesillas-Gutierrez D Martinez M Salinas F Evans A Zilles K Bias between MNI and Talairach coordinates analyzed using the ICBM-152 brain template. *Hum Brain Mapp* 2007; 28: 1194–205. 10.1002/hbm.20345 PMC6871323 17266101

[21] Eickhoff SB Laird AR Grefkes C Wang LE Zilles K Fox PT Coordinate-based activation likelihood estimation meta-analysis of neuroimaging data: a random-effects approach based on empirical estimates of spatial uncertainty. *Hum Brain Mapp* 2009; 30: 2907–26. 10.1002/hbm.20718 PMC2872071 19172646

[22] Eickhoff SB Bzdok D Laird AR Roski C Caspers S Zilles K Co-activation patterns distinguish cortical modules, their connectivity and functional differentiation. *NeuroImage* 2011; 57: 938–49. 10.1016/j.neuroimage.2011.05.021 PMC3129435 21609770

[23] Smith SM Fox PT Miller KL Glahn DC Fox PM Mackay CE Correspondence of the brain's functional architecture during activation and rest. *Proc Natl Acad Sci U S A* 2009; 106: 13040–5. 10.1073/pnas.0905267106 PMC2722273 19620724

[24] Crossley NA Mechelli A Scott J Carletti F Fox PT McGuire P The hubs of the human connectome are generally implicated in the anatomy of brain disorders. *Brain* 2014; 137: 2382–95. 10.1093/brain/awu132 PMC4107735 25057133

[25] Li L Wu M Liao Y Ouyang L Du M Lei D Grey matter reduction associated with posttraumatic stress disorder and traumatic stress. *Neurosci Biobehav Rev* 2014; 43: 163–72. 10.1016/j.neubiorev.2014.04.003 24769403

[26] Javitt DC When doors of perception close: bottom-up models of disrupted cognition in schizophrenia. *Annu Rev Clin Psychol* 2009; 5: 249–75. 10.1146/annurev.clinpsy.032408.153502 PMC4501390 19327031

[27] Orru G Pettersson-Yeo W Marquand AF Sartori G Mechelli A Using support vector machine to identify imaging biomarkers of neurological and psychiatric disease: a critical review. *Neurosci Biobehav Rev* 2012; 36: 1140–52. 10.1016/j.neubiorev.2012.01.004 22305994

[28] Ioannidis JP Excess significance bias in the literature on brain volume abnormalities. *Arch Gen Psychiatry* 2011; 68: 773–80. 10.1001/archgenpsychiatry.2011.28 21464342

[29] Costafreda SG Parametric coordinate-based meta-analysis: valid effect size meta-analysis of studies with differing statistical thresholds. *J Neurosci Methods* 2012; 210: 291–300. 10.1016/j.jneumeth.2012.07.016 22878178

